# Age-specific patterns of lymph node involvement in elderly patients with oral squamous cell carcinoma: a retrospective cohort study

**DOI:** 10.1007/s00784-026-06978-6

**Published:** 2026-06-12

**Authors:** A. M. Schmitz, N. Kern, M. Brauckmann, C. Hoffmann, F. Mrosk, C. Rendenbach, C. Doll, M. Richter, M. Heiland, S. Koerdt

**Affiliations:** Department of Oral and Maxillofacial Surgery, Charité – Universitätsmedizin Berlin, Corporate Member of Freie Universität Berlin and Humboldt-Universität zu Berlin, Augustenburger Platz 1, 13353 Berlin, Germany

**Keywords:** Oral squamous cell carcinoma, Elderly, Lymph node ratio, Lymph node metastasis

## Abstract

**Objectives:**

Age-related alterations in lymphatic and immune function may influence nodal disease patterns in elderly patients with oral squamous cell carcinoma (OSCC). This study aimed to evaluate the association between chronological age and lymph node metastasis (LNM) and to assess whether the lymph node ratio (LNR) retains prognostic relevance for overall survival (OS) across different elderly age groups.

**Materials and methods:**

This retrospective cohort study included 363 patients aged ≥ 65 years who underwent primary surgical treatment for OSCC. Age was analyzed both as predefined age groups and as a continuous variable using natural cubic spline modeling. Associations between age and LNM were examined using logistic regression. The impact of age and LNR on OS was evaluated using Cox proportional hazards models, supplemented by time-dependent receiver operating characteristic analyses.

**Results:**

Chronological age showed no significant linear association with LNM, while spline analyses indicated only a weak non-linear relationship. Higher comorbidity burden was inversely associated with LNM. LNR was not correlated with age and showed comparable distributions across elderly age strata. In survival analyses, LNR demonstrated a strong non-linear association with OS, whereas age exerted only a modest effect. Overall survival did not differ significantly between elderly age groups.

**Conclusions:**

Chronological age alone was not a major determinant of nodal involvement or survival in elderly OSCC patients. LNR remained a robust prognostic indicator independent of age.

**Clinical relevance:**

LNR may aid in risk stratification and postoperative decision-making in elderly OSCC patients, supporting individualized oncologic management beyond chronological age alone.

**Supplementary Information:**

The online version contains supplementary material available at https://doi.org/10.1007/s00784-026-06978-6.

## Introduction

Lymph node metastasis (LNM) is the dominant prognostic factor in oral squamous cell carcinomas (OSCC) and decisively influences surgical and adjuvant treatment strategies [1, 2]. Although histopathological predictors such as depth of invasion (DOI), tumor size, and grading are well established, it remains uncertain whether these parameters retain the same predictive reliability in elderly patients. Age-related changes, such as lymph node fibrosis, reduced lymphatic flow, and immunosenescence, may alter metastatic patterns and challenge conventional assumptions about nodal disease [3–5].

Despite the growing proportion of elderly OSCC patients, age-specific analyses of nodal involvement remain limited [6–9]. Most studies rely on a single, arbitrary age cutoff (commonly 65 or 70 years) to define “elderly” patients, rather than analyzing age as a continuous variable or stratifying into multiple, clinically meaningful age groups. This approach is often driven by statistical convenience, cohort size limitations, and historical precedent, but it fails to capture the biological and clinical heterogeneity within the older population [7, 10, 11]. Comorbidity, a central modifier of immune and inflammatory capacity, is rarely examined alongside age, although both factors may jointly influence metastatic behavior [6, 12, 13]. Functional status, and tumor biology may also modulate metastatic risk within older age groups. Despite this heterogeneity, the literature highlights an overall pattern of a steady decline in lymph node metastasis probability with advancing age, rather than a plateau or non-linear fluctuation [5, 14].

These gaps underscore a central challenge: conventional nodal metrics may not fully account for biological aging, comorbidity, and reduced lymph node yield (LNY) in older patients. This has prompted increasing attention toward measures that inherently normalize for such variability. Among these, the lymph node ratio (LNR), defined as the proportion of metastatic to examined nodes, has emerged as a particularly robust and reproducible prognostic marker that often outperforms traditional pN staging across heterogeneous cohorts [15–18]. Despite age-related changes such as lymph node fibrosis, reduced lymphatic flow, and immunosenescence, which may decrease both lymph node yield and the absolute number of positive nodes, LNR retains its prognostic validity because it normalizes for the total number of nodes examined, thus mitigating the confounding effect of reduced nodal yield in older patients [17, 19, 20]. Studies specifically analyzing elderly subgroups confirm that high LNR is independently associated with worse outcomes, regardless of age or comorbidity burden [21].

Yet, it remains unclear whether proportional nodal metrics such as the LNR maintain their prognostic validity across the continuum of aging in OSCC. In light of age-related alterations in lymphatic architecture, declining metastatic probability, and reduced lymph node yield, it is essential to determine whether age meaningfully modifies the assessment of nodal disease.

Accordingly, this study seeks to delineate age-specific patterns of lymph node involvement using both linear and non-linear modeling of chronological age, and to evaluate whether the LNR offers prognostic information that remains robust and independent of age in elderly OSCC patients.

## Methods

### Study design

**Fig. 1:**
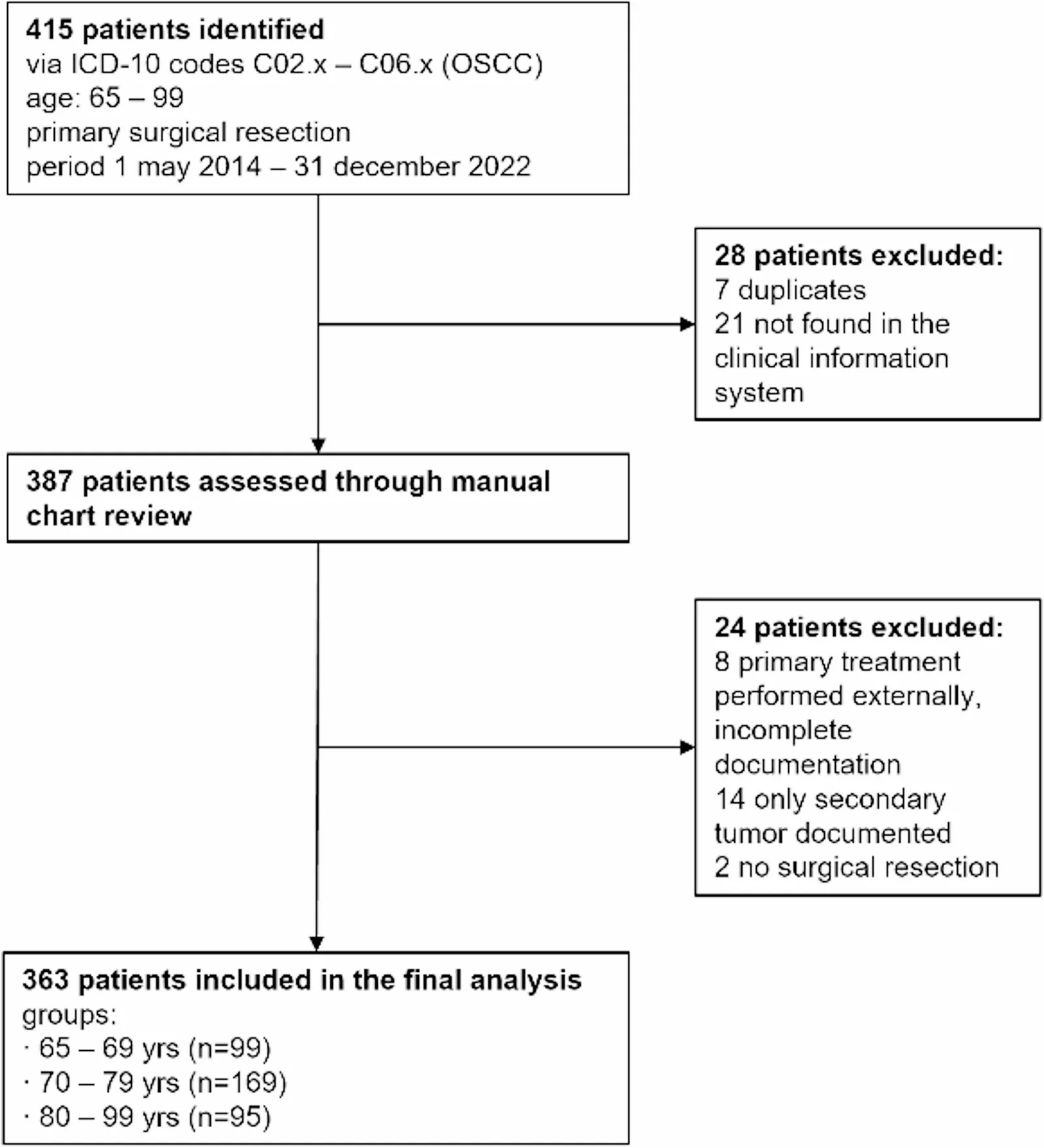
Study flow diagram illustrating patient inclusion, exclusion criteria, and age group stratification

This retrospective cohort study was conducted at the Department of Oral and Maxillofacial Surgery, Charité – Universitätsmedizin Berlin. The institutional database was screened for all patients aged ≥ 65 years who underwent primary surgical treatment for histologically confirmed OSCC between January 2014 and December 2022 (Fig. 1). The study followed the STROBE reporting guidelines and was approved by the institutional ethics committee (EA2-077-20).

### Study population

Eligible patients were identified using ICD-10 codes C02.x–C06.x corresponding to OSCC and were required to have undergone primary curative-intent tumor resection, with or without neck dissection. Neck dissection comprised selective, modified radical, or radical neck dissection, performed according to tumor stage, clinical nodal status, and the German guideline for oral cavity cancer. All histopathological resection statuses (R0, R1, R2, and RX) were included and documented for analysis.

### Age stratification

To examine age-related patterns within the elderly population, patients were stratified into three predefined age groups: 65–69 years, 70–79 years, and 80 years or older. Age was additionally analyzed as a continuous variable to model potential non-linear associations.

### Clinical variables

Demographic and clinical variables included sex, tumor subsite, TNM stage according to the 8th edition, neck dissection, adjuvant therapy, and comorbidity quantified using the Charlson Comorbidity Index (CCI) a validated weighted comorbidity score predicting long-term mortality based on the presence of predefined medical conditions [22, 23]. Histopathological variables comprised tumor grading, depth of invasion (DOI), tumor diameter, presence or absence of LNM, the total number of positive lymph nodes, extranodal extension (ENE), and LNY. The LNR was defined as the number of metastatic lymph nodes divided by the total lymph node count.

### Outcomes

The primary outcome of interest was histopathologically confirmed LNM. Secondary outcomes included LNR, ENE, and overall survival (OS).

### Statistical analysis

All statistical analyses were performed using R (version 4.2.0). Continuous variables were summarized as medians with interquartile ranges and compared using Mann–Whitney U or Kruskal–Wallis tests, while categorical data were analyzed with χ² or Fisher’s exact tests. To evaluate the association between age and the probability of lymph node metastasis (LNM), two logistic regression models were fitted: one treating age as a linear predictor and a second incorporating age as a natural cubic spline with three degrees of freedom. The spline transformation was implemented using the ns() function from the splines package in R, which generates a natural cubic spline basis to flexibly model potential non-linear associations. Default knot placement was used, with no manual specification of knot locations.

Because spline models parameterize predictors using basis functions, the individual spline coefficients are not directly interpretable as effect estimates for specific age comparisons. Therefore, the association between age and LNM was primarily evaluated based on the predicted probability curve across the observed age range. Both models were adjusted for sex, CCI, p16 status, and neck dissection, and the presence of non-linearity was assessed using likelihood ratio testing.

LNR was examined as a continuous variable, and its correlation with chronological age was evaluated using Pearson correlation coefficients. The prognostic impact of LNR on overall survival (OS) was assessed using Cox proportional hazards models including linear and spline terms for LNR, with model performance compared using likelihood ratio tests. OS was estimated using the Kaplan–Meier method, and survival differences between age groups were evaluated using the log-rank test. Time-dependent receiver operating characteristic (ROC) curves at 1, 2, and 3 years were generated to assess prognostic discrimination. A p-value of < 0.05 was considered statistically significant.

## Results

A total of 363 elderly patients met the inclusion criteria, with a median age of 75 years (Interquartile Range (IQR) 70–80; range 65–93). Age groups were distributed as follows: 65–69 years (27.3%, *n* = 99), 70–79 years (46.6%, *n* = 169), and ≥ 80 years (26.2%, *n* = 95) (Table 1).

Among the 333 patients who underwent neck dissection, the median lymph node ratio (LNR) was 4.0 (IQR 3.0–5.0). Patients without neck dissection, including clinically node-negative necks managed without ND, were not included in LNY/LNR analyses.

In this subgroup, the median lymph node yield (LNY) was 24 nodes (IQR 14–37; range 1–109). Stratification by surgical extent showed a median LNY of 22 nodes (IQR 13–34) for ipsilateral and 37 nodes (IQR 24–52) for bilateral neck dissections. When stratified by age group, the median LNY was 29 nodes (IQR 16–41) in patients aged 65–69 years, 25 nodes (IQR 15–37) in those aged 70–79 years, and 24 nodes (IQR 13–36) in patients aged ≥ 80 years. When stratified by nodal status, the median LNY was 23 nodes (IQR 13–32) in pN0 patients and 31 nodes (IQR 22–46.5) in pN+ patients. Using a predefined adequacy threshold of ≥ 10 lymph nodes, 85.6% of patients met this criterion.

Increasing age was positively correlated with comorbidity burden (CCI, *r* = 0.45), whereas chronological age showed a weak inverse correlation with overall survival (*r* = − 0.21).

**Fig. 2:**
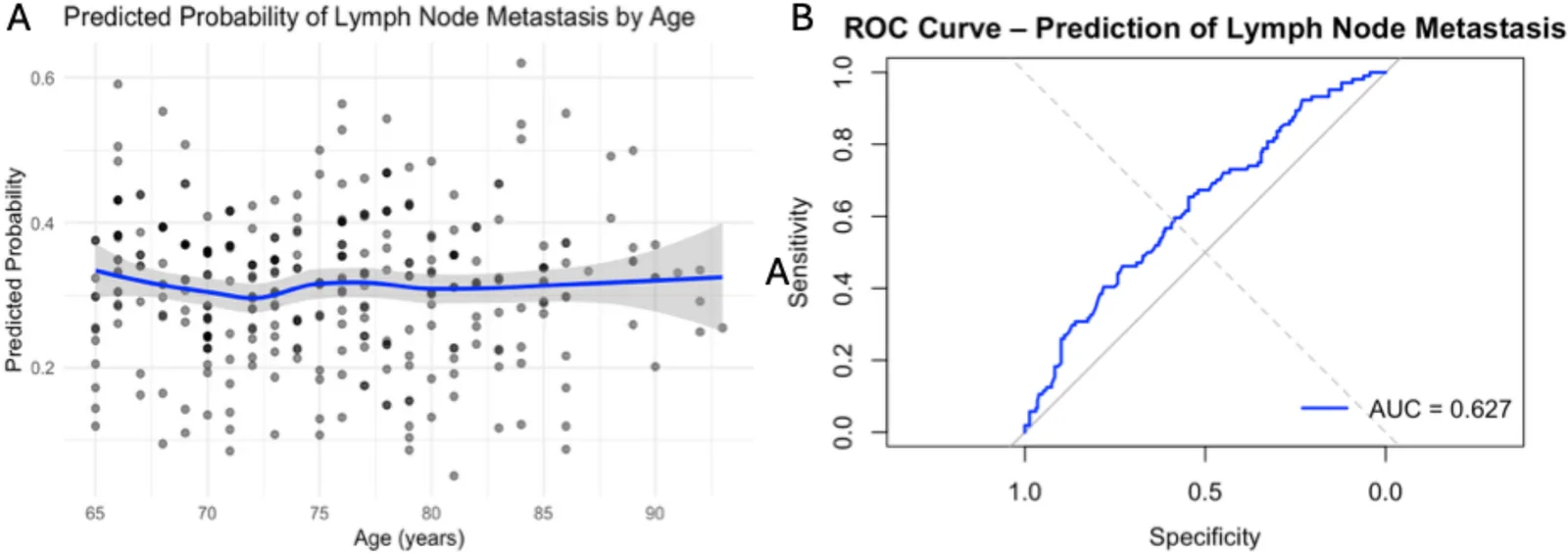
Non-linear association between age and the probability of LNM. (A) Predicted probability of LNM across the observed age range derived from a logistic regression model including age modeled as a natural cubic spline (3 degrees of freedom) and adjusted for sex, CCI, p16 status, and neck dissection. The shaded area represents the 95% confidence interval. (B) ROC curve of age as a standalone predictor, illustrating limited discriminatory performance

In logistic regression analyses treating age as a linear predictor, chronological age was not significantly associated with lymph node metastasis (LNM), and the corresponding ROC analysis demonstrated limited discriminatory ability (AUC 0.55–0.63) (Fig. 2).

**Table 1:** Baseline tumor characteristics and nodal staging stratified by age groups (65–69, 70–79, 80–99 years)

Category	Variable	65–69 years	70–79 years	80–99 years
Tumor Subsite	Floor of mouth	27 (27.3%)	34 (20.1%)	10 (10.5%)
	Tongue	30 (30.3%)	52 (30.8%)	35 (36.8%)
	Mandible	25 (25.3%)	41 (24.3%)	27 (28.4%)
	Maxilla	6 (6.1%)	16 (9.5%)	9 (9.5%)
	Palate	2 (2.0%)	9 (5.3%)	5 (5.3%)
	Buccal mucosa	8 (8.1%)	16 (9.5%)	9 (9.5%)
	Other	1 (1.0%)	1 (0.6%)	0 (0.0%)
Grading	Not specified	6 (6.1%)	13 (7.7%)	5 (5.3%)
	G1	8 (8.1%)	40 (23.7%)	15 (15.8%)
	G2	64 (64.6%)	92 (54.4%)	64 (67.4%)
	G3	21 (21.2%)	24 (14.2%)	11 (11.6%)
	G4	0 (0.0%)	0 (0.0%)	0 (0.0%)
T Stage	pT0/pTx/pTis	2 (2.0%)	1 (0.6%)	3 (3.2%)
	pT1–pT2	52 (52.5%)	98 (58.0%)	53 (55.8%)
	pT3–pT4a-b	45 (45.5%)	70 (41.4%)	39 (41.1%)
N Stage	pN0/pNx	62 (62.6%)	109 (64.5%)	68 (71.6%)
	pN1	11 (11.1%)	20 (11.8%)	10 (10.5%)
	pN2a-c	11 (11.1%)	18 (10.7%)	8 (8.4%)
	pN3a-b	15 (15.2%)	22 (13.0%)	9 (9.5%)
M Stage	pM0	95(96.0%)	163 (96.4%)	83 (87.4%)
	pM1	0	3 (1.8%)	0
Neck Dissection	None	3 (3.0%)	9 (5.3%)	13 (13.7%)
	Ipsilateral	47 (47.5%)	112 (66.3%)	57 (60.0%)
	Contralateral	6 (6.1%)	5 (3.0%)	2 (2.1%)
	Bilateral	43 (43.4%)	43 (25.4%)	23 (24.2%)

When chronological age was modelled as a linear predictor for LNM, no significant association was observed, and discriminatory performance was poor (AUC 0.55–0.63). Modelling age as a natural cubic spline revealed a partially significant non-linear relationship with LNM. The first spline component was strongly associated with LNM (OR 5.05, 95% CI 1.55–16.49), whereas remaining components were non-significant. The predicted probability curve demonstrated a shallow U-shaped pattern across the elderly age spectrum, with moderately higher LNM likelihood in the youngest elderly (65–69 years), lower probabilities in the mid-seventies, and a trend toward increasing rates in the oldest patients (≥ 85 years). Overall, chronological age did not exhibit a strong or monotonic influence on nodal disease in this population.

Comorbidity burden was consistently associated with a reduced probability of LNM. In multivariable logistic regression, CCI remained an independent inverse predictor of nodal involvement (OR 0.79, 95% CI 0.66–0.96), and this association persisted in sensitivity analyses restricted to patients who underwent neck dissection, supporting stability across surgical subgroups.

LNR differed markedly between node-positive and node-negative patients (*p* < 0.001), confirming its validity as a measure of metastatic burden. In contrast to age, LNR showed no correlation with chronological age (*r* = − 0.02), and LNR quartiles were distributed almost identically across all elderly age strata. This indicates that LNR reflects nodal disease severity independent of age.

**Fig. 3:**
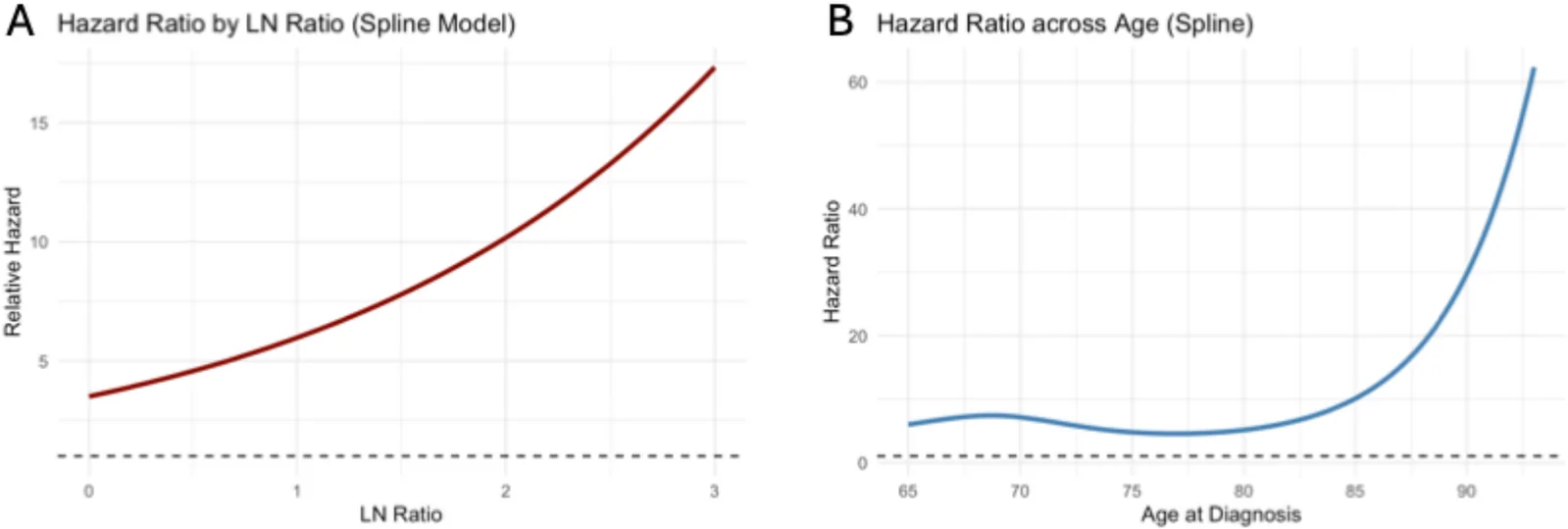
Non-linear association between LN-ratio and overall mortality based on a Cox proportional hazards model with natural cubic splines (df = 3). (A) Spline-based Cox model showing an exponential increase in mortality risk with rising LNR. (B) Spline model illustrating a weak, non-linear association between chronological age and mortality risk across the elderly OSCC population

In Cox proportional hazards modelling, LNR demonstrated a strong non-linear association with OS, and spline-based models significantly outperformed linear models (likelihood ratio *p* < 0.001) (Fig. 3). Mortality risk increased steeply even at relatively low LNR values, resulting in a pronounced risk gradient. In the final adjusted model, chronological age (HR 1.05, 95% CI 1.01–1.09), comorbidity (CCI HR 1.17, 95% CI 1.01–1.35), neck dissection status (HR 3.85, 95% CI 1.53–9.66), and LNR (spline term, *p* < 0.001) were independently associated with mortality, with LNR contributing the strongest effect (Supplementary Table 1).

Time-dependent ROC curves demonstrated increasing discriminative accuracy over time, with AUC values of 0.56, 0.66, and 0.67 at 1, 2, and 3 years, respectively.

**Fig. 4:**
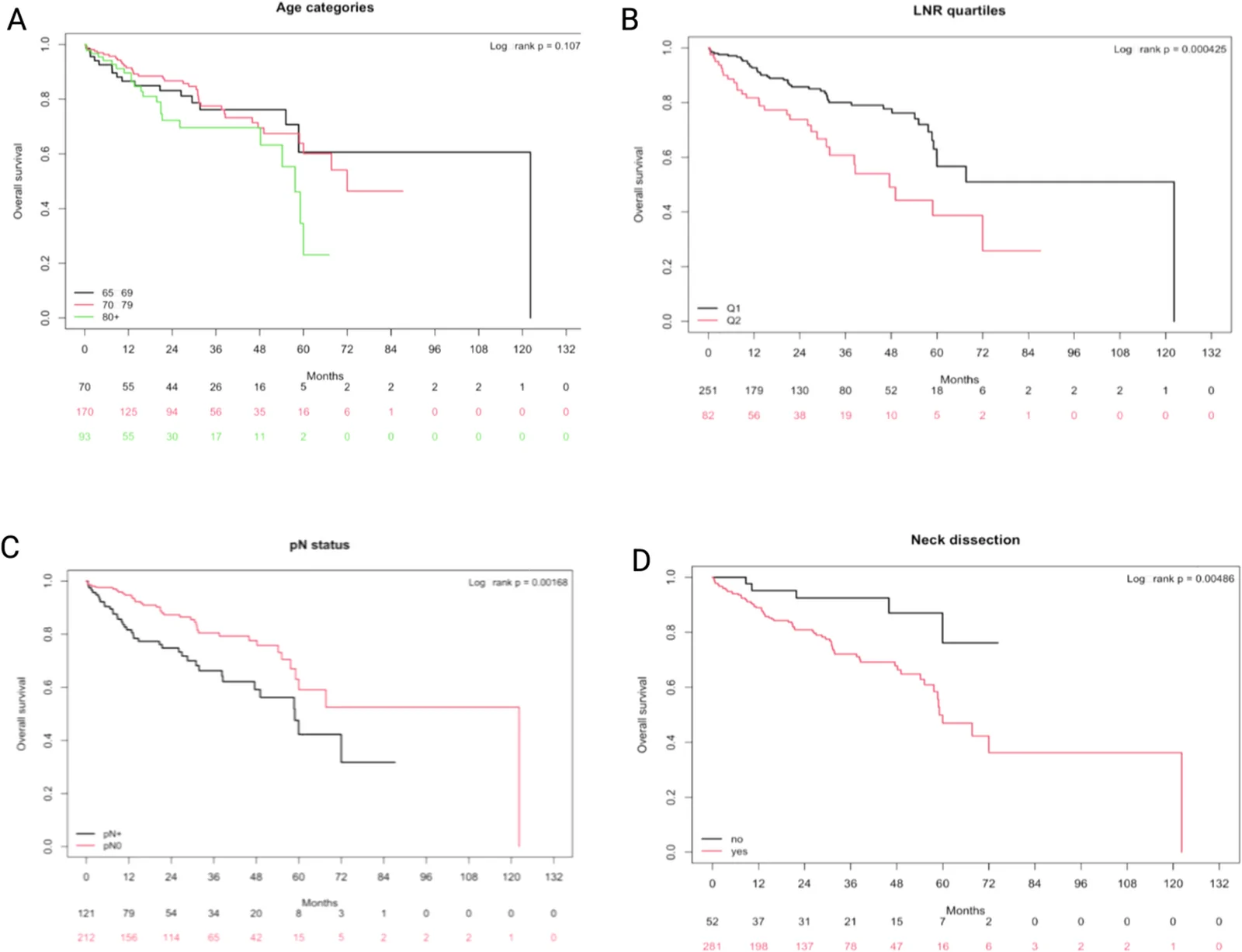
Kaplan–Meier curves for OS across the three elderly age groups (65–69, 70–79, ≥ 80 years). Survival differences did not reach statistical significance (log-rank *p* = 0.059). OS was calculated from the date of primary surgery to death or last follow-up. Time point 0 in the Kaplan–Meier analyses represents the date of surgery and the initial number of patients at risk in each subgroup

Kaplan–Meier curves revealed a trend towards reduced survival across increasing age groups. However, the difference between the three strata did not reach statistical significance (log-rank *p* = 0.059) (Fig. 4).

This indicates that age alone is not a sufficiently strong variable to explain survival differences in elderly OSCC patients, consistent with the weak or non-linear associations observed in LNM modelling.

## Discussion

In this single-center retrospective cohort study, we analyzed age-specific patterns of nodal disease in elderly patients with OSCC and demonstrated that chronological age shows only a weak and non-linear association with LNM. This finding aligns with current evidence that aging affects lymphatic architecture and immune function in a heterogeneous manner, leading to inconsistent metastatic behavior rather than a uniform decline in nodal involvement [11, 24, 25]. Age-related lymph node fibrosis, impaired germinal center activity, and reduced lymphatic transport have been described as mechanisms causing altered or attenuated nodal response in elderly patients, which may explain why the mid-70 age range showed lower LNM probability despite similar tumor characteristics [3, 26–28].

The inverse association between comorbidity (CCI) and LNM observed in our study reflects findings in recent geriatric oncology research, which suggests that systemic illness, frailty, and immunosenescence can suppress lymphatic reactivity and therefore decrease detectable nodal metastasis [11, 29–31]. These effects may lead to under-recognition of nodal disease in multimorbid older adults, independent of surgical extent.

A central and novel finding of our study is that the LNR was entirely independent of chronological age. Prior work has demonstrated that LNR is a strong and biologically meaningful prognostic factor for OSCC, outperforming traditional pN staging and absolute lymph node counts [19, 32, 33]. However, these studies typically included broad age ranges without dedicated analyses of elderly subgroups. Our data extend this evidence by showing that LNR maintains its prognostic value even within an exclusively elderly population, despite known age-related alterations in lymphatic anatomy and immune function. This supports the interpretation of LNR as an age-neutral measure of nodal tumor burden and reinforces its robustness as a biologically grounded marker.

In line with previous literature, LNR emerged as the strongest predictor of OS in our cohort [33, 34]. Recent data further support this, showing that proportional nodal parameters such as LNR and LNY provide strong prognostic discrimination in OSCC, independent of conventional staging categories [35]. The steep, non-linear increase in mortality risk even at low LNR values underscores its clinical significance and suggests that proportional nodal burden captures essential dimensions of tumor aggressiveness that chronological age does not. These findings are consistent with increasing calls in head and neck oncology to integrate proportional nodal metrics into prognostic frameworks and treatment decision-making, particularly in elderly patients where traditional staging systems may inadequately reflect underlying biology [19, 34]. Recent work proposing refined composite nodal measures, such as the MLNC score introduced by Voss et al., which combines the number of metastatic lymph nodes, LNR, and ENE into a composite prognostic metric, underscores this shift toward biologically grounded, proportion-based assessments of nodal tumor burden [32].

The weak and non-significant differences in survival between the three elderly age groups in our cohort support the growing shift from age-based to biologically individualized treatment models [36, 37]. Modern geriatric oncology emphasizes functional status, comorbidity, and tumor biology over chronological age, and our findings reinforce this paradigm by highlighting that advanced age alone should not deter adequate oncologic management [38]. Instead, LNR and comorbidity appear to provide more clinically relevant information for risk stratification and postoperative therapeutic planning [37, 39].

Strengths of this study include the large age-stratified cohort, the use of spline modelling to uncover non-linear age effects, and the comprehensive assessment of nodal metrics. These methodological features address gaps highlighted in recent literature, which has repeatedly called for refined analyses of elderly OSCC populations [10]. Limitations include the retrospective design, the absence of frailty indices, and potential under-staging in the oldest patients due to variability in LNY, challenges that are well documented in head and neck oncology research. Lymph node yield (LNY) may influence the accuracy of nodal staging and the calculation of proportional nodal metrics. In the present cohort, the majority of patients met commonly recommended adequacy thresholds for neck dissection. Nevertheless, a small proportion of cases with lower LNY were included in the analysis. As this study aimed to reflect real-world surgical practice in an elderly population, these cases were not excluded. Importantly, the use of the lymph node ratio (LNR) partially mitigates the influence of variability in nodal yield, as it accounts for the total number of examined nodes. However, potential residual effects of variation in lymph node retrieval cannot be completely excluded.

Future studies should incorporate validated frailty assessments, biological age markers, and multicenter datasets to validate LNR-based prognostic models and to explore whether nodal severity parameters can be integrated into staging systems or decision-support tools specifically for elderly OSCC patients. In particular, combining LNR with geriatric assessments may offer a more accurate prognostic stratification and help guide individualized neck management and adjuvant treatment pathways.

## Conclusions

Chronological age alone should not guide risk stratification or treatment considerations in elderly OSCC patients. Instead, proportional nodal metrics, particularly the LNR, and patient-related factors such as comorbidity offer more meaningful prognostic insight. Incorporating these parameters into clinical assessment may improve individualized decision-making beyond what age-based approaches can achieve.

## Electronic Supplementary Material

Below is the link to the electronic supplementary material.

Supplementary file 1 (DOCX 8.01 MB)

## Data Availability

Due to the sensitive nature of the data, they are not publicly available but may be accessed from the corresponding author upon reasonable request.
