# Correction to: Age-specific patterns of lymph node involvement in elderly patients with oral squamous cell carcinoma: a retrospective cohort study

**DOI:** 10.1007/s00784-026-07146-6

**Published:** 2026-09-24

**Authors:** A. M. Schmitz, N. Kern, M. Brauckmann, C. Hoffmann, F. Mrosk, C. Rendenbach, C. Doll, M. Richter, M. Heiland, S. Koerdt

**Affiliations:** Department of Oral and Maxillofacial Surgery, Charité – Universitätsmedizin Berlin, Corporate Member of Freie Universität Berlin and Humboldt-Universität zu Berlin, Augustenburger Platz 1, 13353 Berlin, Germany

**Correction to: Clin Oral Invest**

**https://doi.org/10.1007/s00784-026-06978-6**

In the original publication of this article, the name of the fourth author was published incorrectly as “C. Hofmann”. The correct spelling is “C. Hoffmann”.

The original article has been corrected.

The online version of the original article can be found at Clinical Oral Investigations https://doi.org/10.1007/s00784-026-06978-6.

