# Supplementary material for: Age-specific patterns of lymph node involvement in elderly patients with oral squamous cell carcinoma: a retrospective cohort study
Source: Clin Oral Investig. 2026 Jun 12;30(7):288. doi: 10.1007/s00784-026-06978-6 (PMC13263194; doi:10.1007/s00784-026-06978-6)
Supplement: Supplementary file 1 — (DOCX 8.01 MB) [file 784_2026_6978_MOESM1_ESM.docx]

**Supplementary Table 1.** Multivariable Cox proportional hazards model for OS. LNR was modelled using restricted cubic splines (3 degrees of freedom).

| Variable | HR) | 95% CI | p-value |
| --- | --- | --- | --- |
| Age (years) | 1.05 | 1.01–1.09 | 0.011 |
| Charlson Comorbidity Index (CCI) | 1.18 | 1.02–1.36 | 0.029 |
| Neck dissection | 3.76 | 1.49–9.47 | 0.005 |
| Lymph node ratio (LNR), spline term | — | — | <0.001* |
|  |  |  |  |

Model performance

| Statistic | Value |
| --- | --- |
| Concordance index (C-index) | 0.625 |
| Likelihood ratio test | χ² = 28.84, p < 0.001 |
| Wald test | χ² = 26.67, p < 0.001 |
| Score (log-rank) test | χ² = 28.67, p < 0.001 |
|  |  |

*Overall significance of the non-linear spline term assessed by likelihood-ratio comparison of spline and linear models.

Abbreviations: HR, hazard ratio; CI, confidence interval; CCI, Charlson Comorbidity Index; LNR, lymph node ratio.
